# “Big Data” Approaches for Prevention of the Metabolic Syndrome

**DOI:** 10.3389/fgene.2022.810152

**Published:** 2022-04-27

**Authors:** Xinping Jiang, Zhang Yang, Shuai Wang, Shuanglin Deng

**Affiliations:** ^1^ Department of United Ultrasound, The First Hospital of Jilin University, Changchun, China; ^2^ Department of Vascular Surgery, The First Hospital of Jilin University, Changchun, China; ^3^ Department of Oncological Neurosurgery, The First Hospital of Jilin University, Changchun, China

**Keywords:** metabolic syndrome, big data and analytics, genome and epigenome, pathogenesis and pathophysiology, methodology of computational research

## Abstract

Metabolic syndrome (MetS) is characterized by the concurrence of multiple metabolic disorders resulting in the increased risk of a variety of diseases related to disrupted metabolism homeostasis. The prevalence of MetS has reached a pandemic level worldwide. In recent years, extensive amount of data have been generated throughout the research targeted or related to the condition with techniques including high-throughput screening and artificial intelligence, and with these “big data”, the prevention of MetS could be pushed to an earlier stage with different data source, data mining tools and analytic tools at different levels. In this review we briefly summarize the recent advances in the study of “big data” applications in the three-level disease prevention for MetS, and illustrate how these technologies could contribute tobetter preventive strategies.

## Introduction

Metabolic syndrome (MetS) consists of a cluster of metabolic conditions that occur simultaneously and significantly increases cardiovascular and cerebral diseases (higher risk for heart attack and stroke) and type 2 diabetes (multiple complications affecting the cardio-cerebral system, nervous system and renal function) and potentially many other pathological conditions, including cancer, neurodegenerative diseases, and non-alcoholic fatty liver diseases (increased risk for cirrhosis and hepatic failure) (Wilson et al., 2005; Esposito et al., 2012; de A Boleti et al., 2021; Golabi et al., 2018). MetS has been estimated to affect a large population globally, with a reported incidence of 34% in the US population (Moore et al., 2017) and 20–25% in the adult population of developed countries (Saklayen, 2018). Generally recognized diagnostic criteria include the concurrence of more than two of the following: (I) increased abdominal circumference (waistline of 40 inches or more for men and 35 inches or more for women), (II) low plasma levels of high-density lipoprotein cholesterol (less than 40 mg/dl (men) or under 50 mg/dl (women)), (III) increased values for plasma triglycerides (above 150 mg/dl), (IV) elevated blood pressure (130/85 mm Hg or higher), and (V) elevated glucose levels (fasting blood glucose level greater than 100 mg/dl), defined by the National Cholesterol Education Program Adult Treatment Panel III (Lorenzo et al., 2007). The current understanding of MetS is a multi-system pathophysiological process initiated by adipose tissue dysfunction and insulin resistance (Després et al., 2008). The former results in the secretion of free acids and a series of cytokines, including tumor necrosis factor, leptin, resistin, and plasminogen activator inhibitor, contributing to insulin resistance (Türkoglu et al., 2003). Its prevalence is high and still increasing, with consequences throughout the body covering the full spectrum of modern diseases. However, the eventual occurrence of these processes is influenced by complex interactions between internal factors such as genetic backgrounds and external factors such as environmental influence, rendering it an extremely heterogeneous pathological entity (McCracken et al., 2018).

When considering intervention for a specific condition, the stage during its natural history directly influences the strategy and options. Prevention corresponding to different stages of diseases has long been promoted as a basic concept for health promotion, with the most commonly adopted “three levels of prevention” being primary (prevent a disease from ever occurring), secondary (early disease detection), and tertiary prevention (reduce the severity of the disease and prevent associated sequelae), with primordial prevention (risk factor reduction) and quaternary prevention (prevention of overmedicalization) having been added later. The prevention of MetS for individuals in a traditional sense would be diet control and exercise counseling in primary, screening for diabetes and cardiovascular diseases in secondary, and cardiac rehabilitation and diabetes complication prevention in tertiary prevention and minimizing side effects from medication in quaternary prevention. However, the mass application of mathematical models and computational tools in medicine has shifted the understanding of disease mechanisms and intervention strategies (Saberi-Karimian et al., 2021). Simultaneously, these applications have generated a vast amount of data from molecular to behavioral levels; with the advent of the concept of “big data,” the scale effects are beginning to reshape clinical paradigms to a degree of unprecedented precision. For MetS prevention and intervention, incorporating “big data” approaches has pushed the overall three-level prevention to an earlier stage with high-throughput technology, machine learning and deep learning, and the latest medical databases and databank. This narrative review summarizes the latest advances (searched on PubMed with key words: “Metabolic syndrome,” “T2D,” “Big data,” “machine learning,” “artificial intelligence,” “deep learning,” “genomics,” “epigenomics,” “metabolomics,” and “proteomics”) in the field to illustrate the applications of common “big data” approaches in the three levels of prevention for MetS.

## Common Data Sources and Analytic Tools for Big Data Research in MetS

The term “data mining” has been given a greater interest in recent years with both reductions of the cost of high-throughput analysis generating “omics” data in the realm of bio-informatics, and the maturation of artificial intelligence in processing data of mixing types from clinical practices (Gonzalez et al., 2016; Lan et al., 2018). The purpose of data mining in itself is to discover patterns in large data sets to generate new knowledge. For this purpose, a group of tools from multidisciplinary fields are available, including database technology providing a source of data, such as the Swedish Longitudinal Integrated Database for Health Insurance and Labour Market Studies (LISA, Longitudinell Integrationsdatabas för Sjukförsäkrings-och Arbetsmarknadsstudier) and diabetes mellitus database (Ludvigsson et al., 2019; Kohsaka et al., 2021), statistics as the mathematical basis, such as the TREAT model, MAYO Clinic model, and Liverpool Lung Project model (Shipe et al., 2019), and machine learning, unsupervised learning, unsupervised learning, and deep learning as analytic tools (Handelman et al., 2018; Rauschert et al., 2020). For MetS preventions on different levels, the current research advance has used different analytical algorithms for various data sources.

### “Big Data” From Omics

“Omics” is a concept to expand the achievement from the Human Genome Project with high-throughput technologies for the comprehensive study of DNA, RNA, metabolites, and proteins. With proper mathematical analysis, differences between study populations could be identified to deepen the understanding of diseases. “Omics” approaches commonly used for MetS study include genome, epigenome, proteome, and metabolome, for assessment of disease risk from both internal and external factors and evaluation of response to interventions. However, for genomic and epigenome studies, available sources are usually from blood samples acquired from populations recruited for extensive prospective studies of single centers or multiple centers, national bio-databases, or models from certain institutes (Chitrala et al., 2020; Nuotio et al., 2020; Parisinos et al., 2020). Techniques involving micro-arrays or chips as high-throughput platforms are commonly used for sequencing and analysis of genomic DNA and DNA methylation from peripheral blood leukocytes, and regression-based mathematical models are applied in many of these studies as genomic-wide and epigenome-wide association studies (Murrell et al., 2005; Wilson et al., 2006; Dixon et al., 2007). Different studies might have variations in the details of adoptions of the database, DNA sample processing, and mathematical tools. Still, the core purpose is to capture the inherent traits of genetic variations between individuals from different backgrounds, most commonly presented as single nucleotide polymorphism in a genome-wide association study, and methylation and histone alterations in an epigenome-wide association study, to relate to MetS occurrence and intervention response. Different from genetics scrutinizing single genes, genomics studies stress all genes and their relationships; hence, it is an essential step for MetS study because of the complexity in its pathogenesis. However, there is a difference in association and causality, and further limited by the fact that most SNPs are found in the non-coding areas, which is still not fully understood, genomic studies such as GWAS alone cannot fill the gap between genetic changes and phenotype changes. The discovered loci can only explain a small amount of heritability, that is, there is a large amount of missing heritability that needs to be further explored. GWAS usually associates genotype to phenotype by computational testing. However, direct functional data are required to assign individual genetic changes to the related phenotype. This makes genomic analysis and epigenome analysis a data source and tool for primordial and primary prevention for MetS, to identify individuals with inherent risk factors for close monitoring.

In contrast to genomic and epigenomic studies, functional omics including transcriptome, proteomic, and metabolomic are more dynamic and local, reflecting the complex gap phenotype and the genetic basis (Lindon et al., 2004). Current MetS research focuses more on the proteomic and metabolomic level changes. Compared with genomic and epigenomic analysis, recent proteomic and metabolomic studies recruit relatively fewer samples with smaller-scale studies, as the technology platforms have been developed later and are more costly and, in certain situations, could require more invasive measures for sample acquisition. Proteomics, as the name suggests, is the large-scale study of proteins. As one of the functional ends, proteomics is organ-specific, meaning different tissue have specific proteomics profiles and change over time (Hsieh et al., 2016). In MetS research, plasma proteins are usually selected as the target for study as they theoretically reflect a more systemic condition; other studies have also involved skeleton muscle and adipose tissue (Leggate et al., 2012; Ayoub et al., 2018; Gueugneau et al., 2021). Available techniques to analyze proteins on an “omics” level include a series of modalities such as micro-array, mass-spectrometry, X-ray crystallography, and nuclear magnetic resonance (NMR) spectroscopy, and with regression models or machine learning algorithms, a proteomic-wide analysis could be conducted (Low et al., 2019). Like proteomics, metabolomics is another functional end of the study and intimately related to proteomics as proteins constantly degrade into metabolites and metabolites participate in post-translational modifications of proteins. Compared with the proteome, metabolome sampling is relatively less invasive as many metabolites could be detected in plasma and urine using standard techniques of NMR or mass-spectrometry (Letertre et al., 2021), making it a more convenient data source and tool for screening of MetS. Data acquired from proteomic and metabolomic studies could potentially present information for ultra-early detection of MetS before meeting the diagnostic criteria and evaluating the response to intervention; hence, they could be powerful tools utilized in secondary and tertiary prevention for MetS.

### “Big Data” From Clinical Practice

Clinical practice contains rich data sources for MetS prevention, with its abundance and heterogeneity and the exponentially growing storage and linkage, and it represents a typical example for big medical data (Obermeyer and Emanuel, 2016). Traditionally, evidence-based medicine regards randomized controlled trials as the evidence source close to the top of the evidence pyramid. However, with the generation of big data in the information age of medicine, alternative sources for clinical evidence such as real-world data, defined by the FDA as “data related to healthcare status, routinely collected from a variety of sources, outside of randomized clinical trials,” have received growing recognition. For MetS research and prevention, these data would come from multiple sources of entirely different dimensions, which could include: *1*) Anthropometric data: height, weight, BMI, body fat percentage, lean body mass, water content, total muscle mass, bone mass, etc., *2*) medical imaging: CT, MRI, ultrasound, etc., *3*) lab test results, and *4*) natural language in electronic health record. Currently, with the development of micro-sensors and chips, the concept of traditional practice and health care have been merged and pushed to new boundaries with technology assemblies such as the internet of things. Objects of everyday use such as vehicles, watches, mobile phones, and health-monitoring devices, when equipped with connectivity for computation, could generate continuous data.

Again, all these data from different dimensions would contain knowledge aiding MetS prevention in ways not previously known. One of the most essential sets of tools to depict these unknowns is data mining technology, characterized by the application of data extracting and analyzing tools such as machine learning to discover patterns among a vast amount of confounding factors, while comparing the utility and efficacy of different algorithms. Machine learning originated as a branch of artificial intelligence to execute automated knowledge acquisition with existing experiences. Roughly categorized into supervised (learning algorithms based on labeled datasets related to outcomes, to predict outcomes with given data) and unsupervised (self-organized algorithms not needing labeled datasets, without predefined results, to discover unknown patterns) algorithms, machine learning contains a wide range of models to achieve the purpose of regression, classification, clustering, and association (Saberi-Karimian et al., 2021). For the same purpose, different models could exhibit different efficacy, such as how in supervised machine learning, the sensitivity and specificity vary among different models and detailed studies are needed to designate the most suitable model. For MetS, various algorithms have been tested, and many highlighted the “random forest” as the most appropriate model (Xia et al., 2021; Yu et al., 2021), and studies have also used deep learning tools to analyze data from medical images to further contribute to the prediction of MetS occurrence and outcomes, contributing to secondary and tertiary prevention strategies (Lin et al., 2021a; Pickhardt et al., 2021).

## Identifying Population With Inherent Risk: Genome and Epigenome “Big Data” for Primordial and Primary Prevention of MetS

As a global health issue, metabolic syndrome and its components have a very high prevalence across regions, but its risk could differ among different populations with different gender, genotype, ethnicity, lifestyle, diet, and physical activity. This reflects the complex pathogenesis of metabolic syndrome and its components, being an outcome of the interplay of interacting factors of both genetic and environmental backgrounds, *via* multiple mechanisms including inflammation, oxidative stress, insulin resistance, changes in lipid metabolism, endothelial dysfunction, and many other factors that are not entirely clear. In this sense, for primary prevention, a critical objective is to precisely locate individuals with inherently higher risk for developing MetS, namely, to look for those with specific “birthmarks,” to enable early reduction of risk factors, hence integrating primordial prevention and early intervention before the onset of disease.

The prevalence of MetS has significant variability across regions that could be attributed to many socio-economic factors, including the availability of health care resources leading to differences in environmental exposure. Still, on the other hand, in terms of heritability, research has estimated its contribution to be up to 13–30% for MetS (Joy and Hegele, 2008; Henneman et al., 2010; L. Monda et al., 2010). Hence, both genetic and epigenetic investigations would add to screening healthy populations who are prone to the disorder.

### MetS Risk by Race and Ethnicity

Racial disparities play a significant role in the prevalence of MetS. In multiracial regions such as the United States, Canada, and Singapore, solid pieces of evidence have been provided by epidemiological studies proving the inequality of MetS burden among populations of Hispanics (highest prevalence in the US), white people, and African Americans; Indigenous Canadians (highest majority in Canada), South Asians, Europeans, and East Asians; and South Asians (highest majority in Singapore), Malays, and Chinese (Lear and Gasevic, 2019). On the genetic level, some of the earliest genome-wide studies identified a series of tag SNPs associated with components of MetS with genomic data (Table 1). Aldi T. Kraja *et al*. reported the first result to locate these genetic variants with MetS as a whole. The research recruited genomic data from 22,161 participants of European ancestry for analysis and found 29 common variants associated with MetS or MetS components, and the majority of them were located in genes participating in lipid metabolism (Kraja et al., 2011), the most influential of which included LPL, CETP, APOA5, ZNF259, BUD13, TRIB1, LOC100129500, and LOC100128154. The top three most influential genes (LPL, CETP, and APOA5) are associated with multiple bioactivities such as the generation of lipoprotein lipase, the transferring of cholesteryl esters in HDLC toward TG-rich lipoproteins, and endocytosis of TG-rich particles (Mead and RAMJI, 2002; Su et al., 2018; Morton et al., 2021). The variants of these genes contributing to a certain proportion of MetS occurrence supported the theory regarding dyslipidemia as a fundamental component in the development of MetS. Their direct associations with MetS have been reported in small sample studies for LPL methylation associating with triglyceride concentrations, increased CETP mass for reduced HDL-C, and reduced LDL particle diameter. For APOA5 variants, a recognized player in MetS, it is believed to affect insulin resistance, systolic blood pressure, and triglyceride levels (Sandhofer et al., 2006; Xiao-Yan et al., 2013; Castellano-Castillo et al., 2018). A later study with GWAS for Finnish cohorts (Kristiansson et al., 2012) identified SNP rs964184 in APOA1/C3/A4/A5 to be associated, most significantly, with all 30 of the very-low-density lipoprotein (VLDL) particle TG metabolites, but not with other MetS traits including waist circumference, blood pressure, or glucose. The study also constructed a risk score system based on previously reported data and then confirmed 22 SNP variants, of which five were associated with glucose, nine with HLD, 6 with TG, 1 with systolic blood pressure, and 1 with waist. The risk score system achieved an Odds ratio of 1.55 comparing the highest quartile with the lowest.

**TABLE 1:** SNPs associated with MetS&MetS components from recent GWAS studies in different races and ethnicities.

Race and ethnicity	Number of samples	Identified genes	Identified SNPs	Race or ethnicity specific SNPs
European ancestry	22,161	LPL	rs13702 (LPL)--- HDLC, TG	NA
		CETP	rs9939224 (CETP)--- HDLC, TG	
		APOA5	rs2266788 (APOA5)---HDLC,TG	
		ZNF259	rs2075290 (ZNF259)---HDLC, TG	
		BUD13	rs10790162 (BUD13)---HDLC,TG	
European ancestry (Finnish population)	11,616	APOA1/C3/A4/A5 LRP1B	rs964184 (APOA1/C3/A4/A5)---VLDL, TG, HDL, rs17771092 (LRP1B)---TG, insulin	NA
African ancestry	4,820	CA10	rs73989312 (CA10), rs73989319(CA10)	rs73989312 rs77244975
		CTNNA3	rs77244975 (CTNNA3)--- Waist circumference	
		RALYL	rs76822696 (RALYL)	
		KSR2	rs7964157 (KSR2)--- Systolic BP	
		MBNL1	rs146816516 (MBNL1)--- Systolic BP	
		BAI3	rs9354671 (BAI3)	
		EDEM1-GRM7	rs2061117 (BAI3)	
		LPL	rs149307971 ( EDEM1-GRM7)--- HDL	
		CETP	rs294 (LPL)	
			rs4523270 (LPL)	
			rs2165558 (LPL)	
			rs35237252 (LPL)	
			rs4783961 (CETP)	
Eastern Asian ancestry (Han ethnicity)	1,994	APOA5ALDH2BUD13	rs651821(APOA5)---TG,HDL-C	rs671rs180326
			rs671(ALDH2)---BMI, WHR, SBP and TG in alcohol drinkers	
			rs445925---LDL-C	
			rs180326 (BUD13)---TG	
Eastern Asian ancestry (Korean ethnicity)	24	APOA5	rs662799 (APOA5)---MetS, TG,HDL	rs1260326, s1260333
		GCKR	rs2075291 (APOA5)----TG,HDL	rs1919127, rs964184
		C2orF16	,rs2266788 (APOA5)---TG rs780092, rs780093, rs780094	rs2075295, rs1558861
		ZPR1	rs1260326, rs1260333 (GCKR)---TG	rs4775041, rs10468017 rs1800588
		BUD13	rs1919127, rs1919128 (C2orf16)---TG	rs72786786, rs173539, rs247616
		ALDH1A2	rs603446, rs964184 (ZPR1)---TG	rs247617, rs3764261
		LIPC	rs2075295, rs11216126, rs1558861 (BUD13)---TG	rs708272, rs7499892
		HERPUD1, CETP	rs4775041, rs10468017 (ALDH1A2)---HDL	
		MTNR1B	rs1800588(LIPC)---HDL-C	
			rs72786786, rs173539, rs247616	
			rs247617, rs3764261 (HERPUD1, CETP)---HDL-C	
			rs708272, rs7499892, rs2303790 (CETP)---HDL-C	
			rs10830962, rs10830963 (MTNR1B)---FBG	

However, like many diseases, race and ethnicity factors combine intrinsic genetic variance and cultural diversity and play essential roles in the general risk of MetS development, such as the significant difference in MetS incidence between American Hispanics, Americans, and African Americans (Lear and Gasevic, 2019). This fact determines the necessity of large-scale studies based on genomics and epigenomics targeting populations with generally different genetic backgrounds. Several regional genome-wide association studies covering populations besides those of European ethnicity have been conducted to determine genetic signatures predicting MetS risk. A study performed with African samples discovered a variant of SNP rs73989312 near CA10 increasing MetS risk, which is African ancestry–specific, reflecting a possible role of brain function (CA10 exclusively expressed in the brain) in the development of MetS in this population (Tekola-Ayele et al., 2015). The SNP rs73989312 near CA10 was predicted to alter binding motifs of transcription factor activator protein-2, leading to dysregulation of adipocyte function and regulation alteration of the nervous system in these populations at risk (Susan and McKenna, 2014). Furthermore, the influence of LPL and CETP loci previously identified in European ancestries has also been confirmed in continental Africans, suggesting their more fundamental roles in the development of MetS. Later study with Han ethnicity samples confirmed the east Asian–specific common variant rs671 (ALDH2, participant of alcohol metabolism) associated with MetS and further discovered a novel secondary TG-associated signal at rs180326 on BUD13 (Zhu et al., 2017). BUD13, located in the APOC3/A4/A5 gene cluster on chromosome11q23.3 to be associated with serum lipid components (Lynn-Htet-Htet et al., 2014), ranked as the fifth most influential gene whose mutation contributed to MetS risk in the European population (rs10790162). But whether the novel rs180326 variant is specific to Han ethnicity among the East Asian population remains to be elucidated. Another study covering East Asian ethnicity analyzed SNPs associated with MetS in the Korean population and found 17 SNPs variants relating to MetS components potentially specific to Koreans (6 TG SNPs: rs1260326, rs1260333, rs1919127, rs964184, rs2075295, and rs1558861 and 11 HDL-C SNPs: rs4775041, rs10468017, rs1800588, rs72786786, rs173539, rs247616, rs247617, rs3764261, rs4783961, rs708272, and rs7499892) (Oh et al., 2020), yet the most influential SNP is rs662799, located in the APOA5 gene, as opposed to rs2266788 in the European population. Adding to the demographic value, a study targeted Korean females to identify rs455489 in DSCAM for fasting plasma glucose and rs7115583 in SIK3 for high-density lipoprotein cholesterol (HDLC) as gender-specific risk factors (Kong and Cho, 2019). With the accumulation of research, large-scale replication, meta-analyses, and fine mapping across worldwide populations of ethnically diverse genetic ancestries will reveal more information about the genetic complexity of MetS. However, GWAS study in itself has certain limitations, such as the reliance on pre-existing genetic variant reference panels, while many sequencings of populations of different ethnic backgrounds on a large scale are still not complete, and there is an inability to detect ultra-rare mutations contributing to disease (Tam et al., 2019).

Race and ethnicity exhibit not only different inherent genetic backgrounds but also involve a large proportion of external factors such as dieting habits and physical activity, which, again, could embed their influences into the genetic background (Salas et al., 2021). Studies have revealed epigenetic differences among different races and ethnicities in their risk in certain diseases such as cancer (different incidence and mortality) and certain types of cancers (e.g., triple-negative breast cancer, infection-related liver, stomach, and cervical cancer). Similarly, as a pathology affected by external factors, evaluating the risk for MetS on an epigenetic level would significantly contribute to primary prevention of MetS as epigenetic changes are reversible, allowing more room for intervention. Basic studies have offered evidence of a complex network of reciprocal interconnections during transcription regulations between epigenetic changes such as DNA methylation and histone modifications and expression regulators (Ramzan et al., 2021). These complexities depicted on a population scale have supported the background role of lipid metabolism in the development of MetS as mentioned above. Recent studies on certain races have also revealed racial-specific epigenetic alterations for risk prediction. One of the latest studies compared race-specific modifications in DNA methylation between African Americans and white people and reported methylation in the ABCG1 gene in both races and increased methylation in the IGF2BP1 gene only in whites (Chitrala et al., 2020). ABCG1 belongs to the family of ATP binding cassette proteins that play a mediating role in free cholesterol efflux to HDL and participates in lipid accumulation, and its lower expression in visceral adipose tissue has been reported to be associated with obesity and MetS (Choromanska et al., 2019), and its epigenetic alteration Cg06500161 is associated both with serum triglycerides and waist circumference (Nuotio et al., 2020). IGF2BP1 is a member of a conserved family of single-stranded RNA-binding proteins (IGF2BP1-3) that have an essential role in modulating embryogenesis and participate in carcinogenesis and the development of chemoresistance (Xinwei et al., 2018). The racial-specific IGF2BP1 methylation (cg24876164) has also been confirmed to be correlated with breast and colon cancers, adding to the link between MetS and the development of certain malignancies (Chitrala et al., 2019).

The large-scale applications of GWAS and EWAS in the study of MetS have been the newer field of research; more coverage of different populations is still needed to achieve practical value that could eventually be developed into risk stratification tools to alter the public health strategies. Similar examples have been reported in the genetic stratification of stroke incidence in patients with cardiometabolic disease. The study combined the genetic data from five trials whose subjects consisted of European ancestry to develop a genetic risk score system based on 32 SNPs. This study found in the primary prevention setting that the 32-SNP system could stratify a hazard ratio for the top versus lowest tertile of 1.27 (Moon et al., 2018). Similar tools could be developed for the prevention of MetS. However, in contrast to stroke, MetS risk is more complicated, MetS is more prevalent and heterogeneous and would require larger populations of longer follow-ups, yet considering the significant increase in multiple disease risk after MetS, GWAS- and EWAS-based risk stratification tools could be valuable when incorporated into public health activities such as routine health examinations and the drafting of health care insurance policies.

### Specific Potential Single Biomarkers Identified by Genome and Epigenome “Big Data” Studies

Based on the extensive initial screening of variants and epigenetic alterations, specific targets have been located for detailed research for MetS risk. SID1 transmembrane family member 2 (SIDT2) is a lysosomal integral membrane protein that promotes insulin secretion. In the laboratory setting, SIDT2 knockout resulted in insulin resistance in peripheral tissue by affecting the IRS-1 signal pathway (Qian-Ying et al., 2020). For its effect on MetS risk, a multiple-phenotype GWAS has identified rs7107152 and rs1242229 SNPs of SIDT2 to be associated with metabolic syndrome risk in the Korean population (Marston et al., 2021), increasing HDL and triglyceride levels among metabolic syndrome-component traits. Yet for the same gene, different variants of rs1784042 and rs17120425 were found in the Mexican population, the former showing an overall association with MetS and with low levels of high-density lipoprotein, the latter with Type 2 diabetes risk (León-Reyes et al., 2020). These results further support the critical role of specific genes linking lipid metabolism and metabolic syndrome, whose variants might have a higher impact among others in risk prediction tools to design.

Somatostatin is a ubiquitous peptide exerting a wide range of actions, including intestinal absorption regulation, gastrointestinal motility, and insulin and glucagon production (Stadaas et al., 1978; Saksena et al., 2009; Vergari et al., 2019). It is the most effective inhibitor of growth hormone (GH) release. In one study, somatostatin was designated as a target for hypertension and hyper-glycerolemia with linkage analysis following genome-wide microsatellite marker scan of 38 families. For blood pressure modulation, the polymorphism of poly-T repeat sequence (rs34872250) in its promoter is associated with higher blood pressure with longer repeats in overweight and obese carriers (Tremblay et al., 2016). A recent study further revealed the association between the rising number of poly-T repetitions with increased MetS onset (Tremblay et al., 2018). Unlike many other potential biomarkers for MetS mainly associated with lipid metabolism, longer poly-T repeats in somatostatin were associated with higher incidence for all MetS components, with a significant increase in the risk of hypertriglyceridemia and low HDL-cholesterol level in men and abdominal obesity, hypertension, and hyperglycemia in women.

On the epigenetic level, an early study designated SOCS3, a gene involved in leptin and insulin signaling, and found that its methylation reduced the risk of MetS and its component, whose effect was also confirmed in later studies for protection against adverse cardiometabolic effects of obesity (Ali et al., 2016). This protection against MetS risk could be due to the increase in the response to the action of leptin and insulin and the reduction in the signaling of IL-6 and other inflammatory cytokines, which have been proven in the laboratory setting with animal subjects undergoing SOC3 methylation or neuron-specific SOC3 knockout (Babon et al., 2012). CPT1A is a transferase involved in the regulation of insulin-mediated inhibition of glucose production, insulin secretion, glycogen synthesis, and appetite control (Zammit, 2008). Early EWAS study found two methylations of CPT1A locus, cg00574958, and cg17058475, as potential MetS risk markers (Das et al., 2016). A later study further confirmed cg00574958 bridging the effect of carbohydrate and fat intake on MetS components, where carbohydrate intake increases cg00574958 methylation, reduces CPT1A expression, and decreases MetS risk, while fat intake acted inversely (Lai et al., 2020).

These targets, with further studies in different populations, could potentially become valuable biomarkers to facilitate MetS risk assessment on a large scale. However, the interpretation of risk assessment with these markers should remain cautious as different markers represent different components of MetS with the additional strength of associations in other populations. Further research is needed to focus on their assemblies and impacts in more integrated systems.

## Early Detection of MetS: From “Omics” to Clinical “Big Data” in Secondary Prevention

The different concept between primary and secondary prevention lies in the difference between “risk” and “early stage.” An individual with certain genetic or epigenetic risk factors does not necessarily develop MetS even without intervention but would undoubtedly benefit from monitoring of early signs of disease development. A current secondary prevention strategy for MetS includes monitoring lipid levels every 6 weeks and serum aminotransferase and CK levels every 6 months. Blood pressure, blood glucose, and HbA1c should be monitored every 3 months. However, ideal secondary prevention of MetS would be capable of identifying the population with or without hereditary risk factors or environmental risk factors for early detection of biological alterations (Larsen et al., 2018) and certain combinations of these alterations (Srikanthan et al., 2016), which could potentially stand out among a vast amount of confounding factors and eventually contribute to the development of MetS. This requires a different methodology compared with the ones used currently, which heavily rely on known factors whose changes already represent the possible occurrence of MetS. The nature of MetS being a systemic disorder determines its analysis, which must consider both patterns of changes of known factors and factors that are unknown and awaiting discovery. This very particular challenge generated the necessity of the merging of the “big data” method with secondary prevention on different levels, to fill in the gap between “early signs” and “risks” and connect primary prevention and secondary prevention as a continuum. For this purpose, biomarkers discovered from functional levels of omics could provide more information regarding the purpose of finding “the early signs.” Several examples have shown this possibility.

### Omics Data From a Tissue Sample

Skeletal muscle has long been confirmed to be related to metabolism with its role as an energy and amino acid reserve and as a significant site for fatty acid oxidation, carbohydrate metabolism, and heat homeostasis (Stump et al., 2006; Baraibar et al., 2013). Skeleton muscle mass has been inversely associated with MetS development in retrospective studies (Park and Yoon, 2013; Kim et al., 2018). At the same time, it is also a location of pathogenesis, such as glucocorticoid receptor expression, micro-vasculopathy, accumulation of intramyocellular lipid droplet, inflammation, and insulin resistance (Whorwood et al., 2002; Petersen et al., 2007; Goodwill and Frisbee, 2012; Marette et al., 2014; Gueugneau et al., 2015).

Muscle loss due to aging is a significant factor influencing the increased MetS risk (Dominguez and Barbagallo, 2016). However, the latest “omics” study suggested this association might not be equivalent to a direct causal relationship. Analysis of muscle specimen for proteomic and transcriptomic profiling between healthy aging and aging with metabolic syndrome revealed a clear distinction of genes differentially regulated by metabolic syndrome and aging, with the former over-expressing genes related to biological processes of cell death and adhesion, ECM and angiogenesis, catabolic process and signaling, under-expressing genes related to electron transport chain, and, on the protein level, a tendency for alterations in NADH/NAD+ shuttle and β-oxidation compared with the fast-to-slow transition and downregulation of glycolysis seen in healthy aging (Gueugneau et al., 2021).

For specific MetS components, a recent study delved into the detailed process from prediabetes into type 2 diabetes to delineate proteomic changes. The study recruited 148 male subjects (20–70 years) of different glucose metabolic statuses from standard glucose tolerance, impaired fasting glucose, and impaired glucose tolerance to T2D. Muscle samples were obtained from the vastus lateralis muscle for mass spectrometry analysis. Under multi-linear regression, the proteome analysis designated 200 proteins for glucose tolerance status (Öhman et al., 2021), including mitochondrial energy metabolism proteins of isocitrate dehydrogenase [NAD] subunit beta, mitochondrial (IDH3B), NADH dehydrogenase [ubiquinone] 1 alpha subcomplex subunit 10, mitochondrial (NDUFA10), and ATP synthase subunit an (MT-ATP6), which were decreased by worsening of the glucose tolerance status and independent of the subjects’ age. Of these decreased proteins, the study also found NADH dehydrogenase [ubiquinone] 1 beta subcomplex subunit 3 (NDUFB3) and NADH dehydrogenase [ubiquinone] 1 alpha subcomplex subunit 2 (NDUFA2) to be increased with physical activity, whose implication highlights the importance of early intervention.

The transition of laboratory studies of omics data acquired from skeletal muscle to preventive medicine practice would require more standardization of tissue acquisition and analysis protocols. Studies have been pushing the refinement of these protocols and the establishment of a database and databank for skeleton muscle proteome and transcriptome analysis (Vissing and Schjerling, 2014; Gonzalez-Freire et al., 2017; Asplund et al., 2020; Deshmukh et al., 2021). Yet challenges remain in certain aspects, such as the representative power of the selected specimen for the metabolic status of an individual as a whole. In contrast, harvesting specimens at multiple locations becomes more invasive.

### Omics Data From a Blood Sample

Compared with muscle specimens, whose harvesting process is relatively invasive and inconvenient, the acquisition of blood specimens is more routinely adopted as a screening approach in clinical practice and studies with large populations. The identification of biomarkers with blood samples is, hence, theoretically of a lower threshold for mass clinical application. An example is beyond the GWAS from the Korean Genome and Epidemiology Study. The project itself accumulated an extensive database for further studies of known biomarkers for MetS risk prediction. Based on this database, several common markers for various conditions, including Gamma-Glutamyl transferase, Adiponectin, Leptin, fasting glucose, and glycated hemoglobin, have been reexamined for their patterns associated with MetS (Jung et al., 2019; Lee et al., 2019; Lee and Shin, 2020).

Besides known markers, metabolomics and lipidomic profiling have been adopted to search for novel patterns as biomarkers. For the former, with simultaneous measurement of metabolites, a large amount of data reflecting the cellular activity at a functional level could achieve a more direct evaluation of the health status. The earliest proof of concept with metabolomics for MetS prediction was a case-control revealing subtle phenotypic differences 5 years before MetS occurrence (Pujos-Guillot et al., 2017). The study applied an untargeted metabolomics approach to identify metabolites from the comparison between normal controls and subjects defined as “pre-MetS” (within normal range but higher BMI, waist-to-hip ratio, systolic and diastolic blood pressure, and fasting blood glucose levels). A 58-metabolite (49 identified with two-way analysis of variance and nine identified with random forest) model was constructed and confirmed to be robust in predicting MetS with the T2D component (all the subjects for model training presented the T2D piece of MetS), from which a linear logistic regression also identified a 5-metabolite model (glutamic acid, phenylalanine, glucose, deoxyglucose phosphate, and L-GPC) that presented similar predictive performances. Interestingly, this study, from a different perspective, further strengthened the feasibility for continuous monitoring of the progression of the T2D component in MetS, which could also be illustrated by the proteomic changes in the skeletal muscle as mentioned previously. Whether this evolution of molecular changes could be captured likewise for other MetS components, or if these changes occur with similar chronological patterns, remains to be studied.

A retrospective study demonstrating the application of metabolomics in MetS used principal component analysis and orthogonal projections to latent structures and achieved a reduction to 26 metabolites involving intrinsic pathways of the urea cycle, amino acid, sphingo- and glycerophospholipid, and sugar metabolisms and metabolites reflecting environmental factors of nutrition, microbiota, and physical activity (Surowiec et al., 2019). Among these metabolites, the study further confirmed the association between higher levels of branched-chain amino acids (with alanine being the strongest association) and high uric acid levels with the risk of T2D and discovered the positive correlation of acylcarnitines with MetS risk. On the other hand, similar to metabolomics, lipidomic analysis, with high-throughput techniques using small volumes of serum or plasma, is capable of identifying and measuring blood plasma levels of hundreds of different lipid species from dozens of different classes and subclasses (Zhao et al., 2020) that have been proven to assert evaluative value for MetS. In one of the studies, Xiaoyan Yin et al. analyzed the lipid profile measured from 658 participants and found 39 lipids associated with obesity and dysglycemia (Yin et al., 2020). Another study covered both metabolomic and lipidomic data for targeting MetS and found 100 lipids of triglycerides, phosphatidylcholines, phosphatidylinositols, and ceramides to correlate with the metabolic syndrome score positively (Surowiec et al., 2019).

However, despite the versatility of high-throughput techniques such as metabolomics and lipidomic analysis, with blood samples being a less invasive method in omics study, the current understanding of metabolomic and lipidomic changes in both pre-MetS and MetS conditions is preliminary. It is obvious that the results from different studies do not correspond well, such as how glutamic acid and phenylalanine in the pre-MetS prediction model did not reach statistical significance in the MetS association study retrospectively. Further application of these techniques in ultra-early diagnosis would require more standardization of research design and analytic tools.

### Multi-Omics and Machine Learning

The future direction of the biomarker identification of MetS, supported by the latest studies, would be the integration of these omics data acquired simultaneously for analysis on a more general level, especially the combination of bio-molecules of metabolites, lipid, and protein which are internally related in the biological system (Whorwood et al., 2002). Some of the latest attempts have gained progression in the integration of data within the realm of metabolomics and lipidomics. A study established a multiplatform of reversed-phase LC-MS (C18) analysis complemented by hydrophilic interaction chromatography (HILIC) to allow the detection of polar metabolites and an untargeted lipidomics approach using a reverse-phase LC-MS (C8) to profile a large set of lipid species. The platform was designed to maximize the serum metabolome coverage (Comte et al., 2021).

However, the processing, interpretation, and integration of large datasets remain a challenge, especially for traditional bioinformatic tools. Hence, new statistical and computational algorithms for data integration, filtering, and network analysis are needed to process extensive multivariate data (Suravajhala et al., 2016; Auerbach et al., 2018). As mentioned previously, machine learning algorithms, with automation features in searching patterns, have been gradually incorporated into the study of ultra-early detection of MetS. An earlier study proved the feasibility of applying machine learning algorithms to simultaneously process metabolomics, lipidomics, and clinical data with a random forest algorithm (Acharjee et al., 2016). A later study further supported the strength of machine learning processing omics data in the realm of MetS, collecting one of the most extensive transcriptomics data to discover nine hub-gene features (SPTAN1, KCTD7, PSMD1, FZD1, KLHL9, PTTG1, TSPAN14, P2RY2, and CXCR5) with excellent classification ability (Liu et al., 2021). With improved coverage of omics data over different populations, machine learning could potentially standardize molecular screening markers for MetS while deepening the understanding of its biological mechanism.

### Clinical Data and Machine Learning

A limitation of molecular traits for secondary prevention for MetS, similar to genome-based studies, lies in the causal relationship. Available omics data and common general biomarkers, on the one hand, could be a direct reflection of the genetic basis, through complex biochemical processes, while on the other hand, succumb to tremendous influence from external factors. Inversely, external factors could assert a broad impact beyond the current technical coverage of detection for functional players such as metabolites and lipids; these components are not yet known and are detectable when they remain in the black box. Therefore, it is not reasonable to regard multi-omics data as the only tool for secondary prevention in the realm of precision medicine. External factors, when facing a black box of pathological processes, the unknowns could be, to a certain level, bypassed with data “big” enough by mathematical and computational tools such as machine learning, and play important roles at a later stage in secondary prevention.

Indeed, many studies of machine learning in metabolic disorders, including MetS, type II diabetes, and hypertension, focus on mining data from extensive medical documentation such as electronic records and medical images from daily practices and large-scale trials (Lin et al., 2021b; Yu et al., 2021). Common data types would include anthropometric data, laboratory tests covering different systems, and medical imaging of other modalities such as x-ray based, ultrasound-based, and even MRI-based techniques. With large enough data, unrecognized patterns could be captured and reveal novel risk factors. The inclusion of many co-variants for study and analysis could have tremendous costs under traditional paradigms (Vrbaški et al., 2019). Machine learning tools are an ideal option to contribute to higher efficiency and precision in identifying the population at risk for developing metabolic disorders for early prevention and those with metabolic diseases with higher risk for a worse prognosis for more extensive management.

A direction of study is to discover the potential expansion of diagnostic criteria at a later stage of secondary prevention. As machine learning is inherently capable of processing a vast number of co-variants, the boundary between the population with or without metabolic diseases might be obscured by the identification of risk factors that were not considered previously for evaluation.

Several studies have reported the application of machine learning for MetS risk/predication with clinical data (Table 2). Cheng-Sheng Yu et al. reported the comparison of different decision tree algorithms over medical data from self-paid health examination by 1,333 individuals, discovering the CAP score (a liver steatosis score), obesity, and HbA1c as being the principal factors predicting metabolic syndrome, and argued the narrowness of the current diagnostic criteria for metabolic syndrome omitting hepatic and nephritic presentations (Yu et al., 2020). Liver steatosis is a condition characterized by increased liver fat content. In terms of nonalcoholic fatty liver disease (NAFLD), it has been reported that despite the prevalence of NAFLD being 18.2% (95% CI 16.5–19.9), it was significantly greater (43.2%) in those with MetS (OR 11.5, 95% CI 8.9–14.7). It increased with the number of MetS criteria (67% for those with all five standards) (Jinjuvadia et al., 2017). Yet the causal relationship between MetS and NAFLD is complicated. MetS and NAFLD share predisposing risk factors such as overeating and physical inactivity. For NAFLD, the pathogenesis includes the promotion of synthesis of intrahepatocellular triglycerides and VLDL. At the same time, the ability of insulin to suppress glucose and VLDL production in the fatty liver is impaired, resulting in mild hyperglycemia and stimulation of insulin secretion, hyper-triglyceridaemia, and low HDL cholesterol concentration, which are also components of MetS. However, it has been reported that carriers of the PNPLA3 Ile148Met allele have an increased risk of the disease but do not typically display features of metabolic syndrome. In a later study, the authors further identified patients with different combinations of specific metabolic traits and especially potential patients in the non-obese population (Yu et al., 2021), and with random forest algorithm, further confirmed the capability of the three parameters, namely, CAP score, HbA1c, and body mass index (study focusing on non-obese patients), in predicting MetS in a 3-year follow-up. This is an example of the versatility of data research in shifting the traditional diagnostic concept of diseases, especially with continuous variables such as CAP score to replace an arbitrary diagnosis of NAFLD, reflecting a progressive development of MetS. Yet these studies have not answered the integration of diagnostic parameters for better prediction.

**TABLE 2:** Machine learning for MetS risk/prediction with clinical data.

	Data source	Sample size	Studied machine learning tools	Clinical data type	Optimal algorithm	Associated/significant clinical index
Retrospective Cohort, Cheng-Sheng Yu, 2020	Health examination	1,333	Decision tree---classification and regression trees, C5.0, chi-square automatic interaction detection, conditional interference trees, evolutionary learning of globally optimal trees, generalized linear model trees, random forest	Anthropometrics, laboratory tests, medical imaging	NA	Obesity, serum GOT, serum GPT, CAP score, HbA1c
Cheng-Sheng Yu, 2021, Retrospective Cohort+3 year follow-up	Health examination	1,129	K-nearest neighbor classification (KNN), linear discriminant analysis (LDA), logistic regression for classification, ensemble learning:random forest, adaptive boosting, support vector machine (SVM), naive Bayes classification (NB), and hierarchical clustering analysis (HCA)	Anthropometrics, laboratory tests, medical imaging	Random forest	Body mass index, HbA1c, CAP score
Ji-Eun Park, 2021, Retrospective Cohor	Korean Genome and Epidemiology Study	2,871	K-nearest neighbor (KNN), naive Bayes, random forest, decision tree, multilayer perceptron (MLP), support vector machine (SVM)	Anthropometrics, life style data	Naive Bayes (most sensitive)	Age, stress (potential predictors included age, sex, education level, marital status, body mass index (BMI), physical activity, alcohol consumption, and smoking)
Shu-jie Xia, 2021, Retrospective Cohort	In-patient	586	Decision tree (DT), support vector machine (SVM) and random forest (RF)	Anthropometrics, laboratory tests, TCM indexes	Random forest (RF) (best performance)	Waist circumference, fasting blood-glucose, BMI, alkaline phosphatase creatinine, blood urea nitrogen, AST/ALT, weight, TCM indexes: body fat, wiry pulse, chest tightness, spontaneous perspiration, greasy tongue coating, snoring sleep
Perry J. Pickhardt, 2021, retrospective cohort	HIPAA-compliant investigation	7,785	Convolutional neural network (3D U-Net), region-based convolutional neural network (R-CNN)	CT-based biomakers	NA	Univariate L1-level total abdominal fat** (80.1% sensitivity, 85.4% specificity), L3-level skeletal muscle index, volumetric liver attenuation

A similar study adopted a broader expansion for risk factor inclusion by incorporating concepts from traditional medicine, as conventional medicine has statistical significance *via* long-term accumulation of “medical data.” The study included the “Sasang constitution type” from traditional Korean medicine for comparison of six types of machine learning methods over a data set of 2,871 visitors from a medical center and discovered higher sensitivity for prediction with incorporation “Sasang constitution type” (Park et al., 2021). Another study incorporating traditional medicine methodology combined clinical variables, including 20 physicochemical indexes commonly tested in routine medical practice, with 47 symptoms described within the framework of traditional Chinese medicine (Xia et al., 2021). Three machine learning methods were tested with these data resulting in the superiority of the rain forest model, whose prediction power increased with the incorporation of symptom variables.

Medical imaging plays a crucial role in both preventive and clinical medicine, offering structural and functional information on the health status of different organs and systems. In metabolic diseases, the very early development and progression can leave local traces which might not parallel with systemic changes. Yet, these signals might not be identified by a human observer (Han et al., 2010). Therefore, the mining of imaging data with machine learning tools could provide early signs of both risk and diagnosis from a different dimension. Examples of machine learning in the application of early MetS detection are abdominal imaging. CT and MRI or ultrasound are commonly administered screening modalities for various abdominal diseases both emergent or chronic (Tanaka et al., 2006). This location of imaging harbors indicators including visceral fat, hepatic steatosis, and skeleton muscle, which are closely related to MetS (Lin et al., 2021a; Chou et al., 2021; Pickhardt et al., 2021; Wu and Park, 2021). Machine learning, especially deep learning methods, has been studied for fast automated quantification of different fat compartments, level of steatosis, and fat distribution (Sun et al., 2020; Bhanu et al., 2021; Rhyou and Yoo, 2021). A recent report utilized imaging data from opportunistic abdominal CT scanning from 9,223 adults, with deep learning-based segmentation of image and analysis, and found an L1-level total abdominal fat threshold of 460.6 cm could achieve an 80.1% sensitivity and 85.4% specificity for asymptomatic metabolic syndrome (Pickhardt et al., 2021).

## Detection of MetS Progression: Machine Learning in Tertiary Prevention

MetS is the clustering of cardiovascular risk factors and is known as a powerful predictor of diabetes and cardiovascular disease. MetS portends increased risk for chronic disease and mortality. The most well-known link is between MetS and the incidence of cardiovascular disease. However, there are established links between MetS and increased risk of chronic kidney disease, diabetes mellitus, arthritis, schizophrenia, non-alcoholic fatty liver disease, and multiple types of cancer. MetS is associated with a greater risk of mortality, a 2-fold increased risk for cardiovascular events or death, and a 1.8-fold increased risk of mortality (Gonzalez et al., 2016; Saberi-Karimian et al., 2021). Both metabolic syndrome and its metabolic consequence as type II diabetes could lead to significant health burden and mortality *via* direct and indirect processes. Such unsatisfying prognosis could be encountered in patients without proper management and those with both known and unknown factors.

### Prognosis Prediction

Glycated hemoglobin (HbA1c) is used as one of the diagnostic criteria for diabetes and the category of increased risk for diabetes. A well-known indicator of metabolic disease reflecting the quality of control is glycosylated hemoglobin, representing an average glucose level for the previous 2–3 months. Its status was further confirmed to be a predictive factor, together with fasting plasma glucose and body mass index, for the development of type 2 diabetes in patients of metabolic syndrome (Park et al., 2012). Sang Hyun Park et-al. suggested that HbA1c might be used as a diagnostic criterion for MetS and the appropriate cut-off value of HbA1c may be 5.65% in this Korean population. Machine learning methods to determine the optimal variables should define distinct cohorts of HbA1c trends. Machine learning carries the capability of identifying subtle multivariate relationships and has emerged as a powerful tool in the analysis of large databases. Avivit Cahn et al. explored the association of HbA1c changes and mortality in a National Registry of patients with type 2 diabetes. They reported unstable glycosylated hemoglobin, compared with the stable level, and predicted an increased all-cause mortality rate. The study covered a massive cohort of 293,314 patients. The machine learning method provided evidence supporting the association of HbA1c variability with mortality (Cahn et al., 2022), which had conflicting results in previous studies. In previous studies, the association of HbA1c variability with mortality yielded inconsistent results, largely dependent upon sample size, adjustment for confounders, and measures of variability used. Seven HbA1c SDs were independently associated with mortality only in the older age groups (age over 55). Moreover, HbA1c had a J-shaped association with cumulative mortality, apparent in all age groups. Studies that did not analyze outcomes by age or HbA1c categories may have failed to associate HbA1c variability with mortality due to the inclusion of disparate clinical phenotypes in a single model.

For factors not yet determined, studies have also utilized machine learning in scrutinizing the natural language of clinical notes in predicting mortality rate in clinical scenarios with abundant confounding factors such as critical care in diabetic patients (De Silva et al., 2021). Machine learning has been used effectively for diabetes research, demonstrating its potential for advancing the management of various diabetes phenotypes across their natural histories (Türkoglu et al., 2003; Lorenzo et al., 2007; Després et al., 2008). With the advent of natural language processing—a branch of artificial intelligence amenable to unstructured text data—clinical text mining is increasingly used in various health domains (Lan et al., 2018; Ludvigsson et al., 2019; Kohsaka et al., 2021). In diabetes research, it has been used in areas such as the analysis of protein–protein interactions and early drug discovery (Shipe et al., 2019; Rauschert et al., 2020). Such studies could reveal unknown associations and risk factors besides objective information provided by laboratory tests and medical imaging, providing valuable dimensions for evaluating the progression of metabolic diseases and their eventual outcome.

### Complication Prediction and Early Detection

Metabolic syndrome and its components could contribute to the development of a series of complications leading to microvascular and macrovascular disease, cardiovascular disease, retinopathy, nephropathy, neuropathy, and many other disorders with chronic conditions, especially for those with insulin resistance and the development of type 2 diabetes (Cull et al., 2007; Czernichow et al., 2010; Callaghan and Feldman, 2013; Mbata et al., 2017). These complications remain prevalent especially in areas with weak medical resources, adding health burdens. Early detection of chronic complications and prevention of acute complications would facilitate health management. The application of mathematical tools has been proven effective in specific scenarios.

To prevent acute complications of metabolic disorders, mathematical and computational tools such as machine learning models could utilize the abundance of data from both public databanks and electronic hospital records for risk evaluation. An example is hypoglycemia, a frequently occurring severe complication with the risk of permanent neurological damage and fatality affecting diabetic patients (Frier, 2014). Analyzing de-identified health claims data using machine learning analytic tools from the Bayesian machine learning platform has been reported to successfully identify diabetic patients with prior history and hypoglycemia and anemia as a subgroup with the highest risk for future hypoglycemia events, enabling specific cautions for hypoglycemia prevention in this population (Mueller et al., 2020). Hypoglycemia, a common adverse effect of treatment of diabetes mellitus with insulin and sulphonylureas, is associated with impairment of cognitive function, which can have significant consequences on everyday behavior. Adults with type 1 diabetes mellitus have ∼2 episodes of mild hypoglycemia per week; the annual prevalence of severe hypoglycaemia is ∼30%. Adults with insulin-treated type 2 diabetes mellitus experience a lower frequency of mild and severe hypoglycemia episodes than those with type 1 diabetes mellitus. Hypoglycemia is the most common and feared adverse effect of insulin therapy and is the most significant barrier to the maintenance of near-normoglycaemia. The risk of hypoglycemia creates internal conflict within the individual, diminishing motivation to adhere to intensive therapeutic regimens and attain strict glycemic control despite the knowledge that achieving this goal would minimize the risk of diabetic complications. The elimination of hypoglycemia would make the management of diabetes mellitus much less demanding (Frier, 2014). In the inpatient setting, where hypoglycemia is a substantial risk, a study of the comparison between different machine learning prediction models based on abundant local medical data could contribute to more precise inpatient management. Recent advances using deep-learning classifiers have led to applications of artificial intelligence (AI) in many areas of health care, including image-based diagnosis and natural language processing. In particular, convolutional neural networks (CNNs) with transfer learning have facilitated efficient and accurate image-based diagnosis well beyond human capabilities. By adopting advanced treatment options such as continuous glucose monitoring (CGM) or closed-loop insulin delivery, the patients predicted by the model with high risk could potentially avoid such complications (Ruan et al., 2020). Kang Zhang at el showed that deep-learning models could be used to identify type 2 diabetes solely in combination with clinical metadata (age, sex, height, weight, body-mass index, and blood pressure) (Zhang et al., 2021). The models were trained and validated with a total of 115,344 retinal fundus photographs from 57,672 patients and can also be used to predict estimated blood-glucose levels and to stratify patients according to disease-progression risk. They evaluated the generalizability of the models for identifying type 2 diabetes with population-based external validation cohorts and *via* a prospective study captured with smartphones and assessed the feasibility of predicting disease progression in a longitudinal cohort.

Chronic complications of metabolic syndrome and its components are a significant determinant of the eventual prognosis affecting disease morbidity, mortality, and quality of life. Many of the complications could be challenging to identify at an early stage, and the optimal strategy for those already in the state of metabolic syndrome with or without type 2 diabetes is to capture the objective but indirect signs at the very early stage before the irreversible end-organ damage. This attempt has been reported to be facilitated by algorithms in analyzing the extent of the disease development. A study focused on delineating the relationship between hyperglycemia and skeletal muscle mass. As mentioned above, skeletal muscle mass is an essential part of insulin stimulation, maintenance of glucose homeostasis, and fatty acid metabolism. The study trained seven machine learning algorithms with body composition data from 6,657 participants. Models generated by XGBoost and ANN algorithms showed good accuracy for predicting skeletal muscle mass and found serum platelet levels, triglyceride concentrations, and glomerular filtration rate as related biomarkers and an inverse relationship between hyperglycemia and skeletal muscle mass, enabling the potential for early management with diet control and physical exercise (Wu and Park, 2021). For patients already in the state of type 2 diabetes, another study reported the efficacy of deep learning models for predicting chronic kidney disease, which is an essential comorbidity of diabetes challenged with early diagnosis. The study attempted to find the early signs in retinal fundus changes to indicate chronic kidney disease risk. Deep learning models were trained with imaging data from 115,344 retinal fundus photographs and achieved prediction of glomerular filtration rates with mean absolute errors of 11.1–13.4 ml min^−1^ per 1.73  m^2^, with the potential for a clinical application non-invasive, high-throughput, and low-cost screening tool (Zhang et al., 2021). While the model appears well suited for diagnostic screening, it remains limited in providing prognostic information to individual patients or insights into pathogenic mechanisms based on saliency maps, regarding the feature importance analysis, simple XGBoost, random forest, and linear regression exploring important variables without knowing the impact of each variable. However, according to Lundberg’s research on Shapley Additive exPlanations (SHAPs), the SHAP value can explain how each feature affects machine learning algorithms, providing a new interpretation method for the machine learning black-box algorithms. In a future study, the SHAP algorithm needs to be used to determine the impact of each variable in the prediction model. In addition, whether additional clinical metadata (such as blood pressure trends, smoking status, alcohol consumption level, and family history) could further improve the accuracy of the predictions needs to be explored.

## Interventions for MetS Supported by “Big Data” Approaches

Traditional interventions for MetS focus on preventing progression into type 2 diabetes, the development of cardiovascular diseases, and many neurological complications. Evidence has been provided by numerous studies supporting the role of lifestyle modifications in delaying T2D onset, reducing incidents of cardiovascular events and microvascular complications, and all-cause deaths. Effects on normalizing glucose tolerance, reduction in blood pressure, lipid, and hyper-insulinemia were also observed (Nilsson et al., 2019).

### Diet Control

A significant component of lifestyle modification relies on the conversion of dietary habits. Extensive research has provided evidence of food quality improvement and macronutrient adjustment benefiting MetS intervention. A variety of diet strategies, including the Mediterranean diet, DASH diet, plant-based diets, ketogenic diets, low-fat diet, and high-protein diet have been reported with different improvements of MetS traits (Castro-Barquero et al., 2020). Recent advances of omics studies have also helped with the deepening understanding of the reaction to diet strategies, possibly facilitating more precise dietary control, such as decreased gamma-tocopherol by DASH diet and lowered TMAO and l‐carnitine by plant-based diets (Pourafshar et al., 2021; Wang et al., 2021).

Of the many diets studied for health control, the Mediterranean diet has been given special credit as the best diet since 2018. Mediterranean diet has high-fat and low-carbohydrate pattern, characterized by a high intake of vegetables including leafy green vegetables, fruits, whole-grain cereals, pulses, legumes, nuts, and extra virgin olive oil as the primary source of fat (Critselis and Panagiotakos, 2020). Some omics studies have revealed the details of the mechanism of this diet strategy on MetS control. An early study reported a significant reduction in inflammation-related inter-α-trypsin inhibitor heavy chain H4 by HDL proteomic analysis in subjects consuming 5 weeks of the Mediterranean diet (Richard et al., 2014). This potential anti-inflammation effect partly explains the intervening development of the Mediterranean diet, as pro-inflammatory has later been confirmed to increase MetS risk and its component, though a gender difference in risk was also observed; hence, the details could benefit from the further study (Khan et al., 2020). Another study revealed metabolic changes after the Mediterranean diet for 2 months and 6 months. After 2 months of the Mediterranean diet, a significant reduction in levels of hippuric acid and lipid levels was observed, associated with removal of inflammatory markers, and reduction of palmitic acid and lactic acid and increase in lithocholic acid were observed after more extended intervention at 6 months (Bondia-Pons et al., 2015).

### Nutrient Response Evaluation

When delving deeper into the components of diet patterns and their effects on the MetS population, much attention has been given to dietary protein and fat. Current understanding of diet protein’s participation in MetS intervention focuses on its effect on alteration of glycemic response and glycemic load, by delaying gastric emptying and stimulating insulin, glucagon-like peptide-1, and peptide YY secretion, and outcomes in preventing specific MetS components *via* fat mass reduction while maintaining lean body mass during energy-deficiency and increasing HDL cholesterol (Chalvon-Demersay et al., 2017). One study with metabolome analysis was with pre-meal whey protein to study the metabolic changes of the Staub–Traugott effect (Bonuccelli et al., 2009), which represents the phenomenon of sequential glucose loading improving glucose tolerance. The study used different doses of whey protein and various types of protein, including whey protein, casein, or gluten, as pre-meal with different timing. The analysis indicated the effect of pre-meal protein in elevating BCAA and aromatic amino acid levels, decreasing decreased PC (32:1) and PC (35:2) levels, which had a significant influence on insulin secretion, skeletal muscle glucose uptake, and improving insulin response (Pekmez et al., 2020).

Dietary fat considered for MetS intervention mainly consists of lipids and fatty acids. The former has received long controversies regarding its influence on triglyceride and high-density lipoprotein. Increasing evidence suggests the replacement of carbohydrate with fat, regardless of type, could lead to the reduction of fasting triglyceride and increase HDL cholesterol; however, contrasting evidence exists (Mensink et al., 2003; Siri-Tarino et al., 2010; Itoh et al., 2014). Early studies regarding the molecular influence between different types of dietary lipids found high-saturated fatty acid diet increases proteins responding to oxidative stress and DNA damages on the proteomic level in patients with metabolic syndrome with both short-term and long-term food intake (Rangel-Zúñiga et al., 2015). Furthermore, there was other evidence suggesting the detrimental effect is more conspicuous in MetS patient with insulin resistance trait (Yubero-Serrano et al., 2015). Considering the insulin secretion–inducing effect of a high-saturated fatty acid diet in healthy individuals, further studies with data from different omics could benefit the understanding of the source of dispute for dietary fat for diverse populations. On the other hand, unsaturated fatty acids, commonly from fish oil component, nuts, and dairy product, have been confirmed to have a benefit for lowering metabolic syndrome risk in multiple studies. Still, arguments also exist for their virtual effect on the population (Eslick et al., 2009; Guo et al., 2017). These discrepancies have recently been shed lighter upon with significant data approaches. One example is a study GWAS which found substantial variations of fatty acid desaturase gene in a Mediterranean population with metabolic syndrome. The fatty acid desaturase gene is an important determinant of serum polyunsaturated fatty acids level through its endogenous production *via* a series of desaturations steps. The GWAS found in Mediterranean metabolic syndrome the association of FADS1/FADS2 locus and omega-3 polyunsaturated fatty acids and omega-6/omega-3 ratio, revealing a potential confounder explaining previous inconsistencies (Coltell et al., 2020). As for the effect of fatty acids on a metabolic level, one recent study tested the influence of Omega-3 fatty acids in healthy old subjects and found its supplementation leads to reduction of total triglycerides, diacylglycerols, phospholipids, and triacylglycerols and very-low-density lipoproteins, whose elevations are associated with metabolic diseases (Xyda et al., 2020). Animal studies have confirmed Omega-3 fatty acid provides cell signaling intermediate (specialized pro-resolving mediators) precursors for inflammation resolution in the adipose tissue microenvironment and elevates insulin sensitivity in the condition of MetS (Carracedo et al., 2019; Kwon, 2020). In human trials, it was also reported to assist with facilitate cognitive dysfunction in MetS patients by enhancing brain-derived neurotrophic factors (Tang et al., 2020). More discoveries of acting mechanisms and finding the sub-population of MetS that would benefit from its intake would be unveiled by more big data studies based on omics and other data sources.

### Physical Activity

The positive link between physical inactivity and metabolic diseases has long been recognized, and this link for MetS has recently been confirmed with abundant evidence (Amirfaiz and Shahril, 2018). Furthermore, physical activity which consists of daily physical activity and exercise has been shown to prevent MetS and its outcomes (Kudo et al., 2021). For MetS intervention, increased physical activity (in particular aerobic exercise) has been shown to improve dyslipidemia and insulin sensitivity, and more specifically, resistance training plus aerobic high-intensity interval training improves fasting glucose low density lipoprotein and insulin secretion, while resistance training plus moderate-intensity continuous aerobic training reduces triglycerides (Da Silva et al., 2020). Physical exercises also induces a series of epigenetic modifications including DNA hypomethylation in muscle tissue at PGC1a, TFAM, PPARD, PDK4, RUNX1, MEF2A, THADA, NDUFC2, ADPOR1, ADIPOR2, and BDKRB; altered DNA methylation in adipose tissue with hypermethylation (ADAMT59, CPEB4, GRB14, ITPR2, LY86, LYPLAL1, MAP2K5, MSRA, MTIF3, MRXN3, PRKD1, SOCCAG8, STAB1, TBX15, TMEM160, and ZNF608) or hypomethylation (GPRC58, TUB) in obesity candidate genes; and exercise-induced hypermethylation (ADAMT59, ADCY5, ARAP1, BCL11A, CDKAL1, CDKN2A, DGKB, DUSP8, FTO, HHEX, HMGA2, IGF2BP2, JAZF1, KCNQ1, PRC1, PROX1, PTPRD, TCF7L2, THADA, WFS1, and ZBED3) or hypomethylation (KCNQ1 and TCF7L2) in T2D candidate genes. However, in terms of lifestyle modification, evidence also suggests that the response to physical activity is heterogeneous, and its balance with diet control as an intervention strategy should vary among different individuals. Several genetic factors have been studied to potentially contribute to this heterogeneity including SNPs for FTO, MC4R, PPARγ2, and ADRB3 for central obesity, APOA5, LIPC, CETP, FADS2, APOB, and PGS1 for dyslipidemia, P13K, LIPC, ADRA2B, TCF7L2, PPARγ2, and ENPP1 for hyperglycemia, and ACE for hypertension (Fenwick et al., 2019). Metabolomic studies have indicated positive serum metabolomic changes for MetS patients following exercise including changes in prominent serum biomarkers of metabolic risks, such as branched-chain amino acids, alanine, acetylcarnitine, choline, and betaine (Siopi et al., 2019a). In terms of types of physical exercises, resistance exercise was shown to cause the most potent metabolic responses, followed by high-intensity interval training, and continuous moderate-intensity exercise achieved minimal response (Siopi et al., 2019b).

### Applications in Pharmaceutical Interventions

Pharmaceutical interventions focus on stabilizing certain metabolic traits, as one drug to treat all components is still unavailable. Available options include statin and non-statin lipid-lowering medications for dyslipidemia, antihypertensive medications for arterial hypertension and prehypertension, and metformin for glucose intolerance. Their clinical benefit and timing of administering and target populations are still under extensive research. A direction for further development of pharmaceutical intervention for MetS is predicting individual drug response. Besides conventional studies based on animal models in clinical trials, a high-throughput analysis could implicate more when considering individuals. Such as on the genomic level, GWAS has designated several variants affecting drug response for MetS components, including rs3846662 located on intron 13 of HMGCR for simvastatin, Rs776746 (C/T) variant found on CYP3A5 gene for a statin, and rs12255372 of TCF7L2 gene for sulfonylureas (Jmel et al., 2018). Besides drug response prediction, effective data approaches such as machine learning have also been studied as a preliminary application in finding pharmaceutic combinations not previously recognized based on data abstracted from the electronic health record, drug recommendation *via* machine learning from publications data sources, and drug–drug interaction discovery based on transcriptome data analysis (Koren et al., 2018; Shu et al., 2018; Luo et al., 2021).

## Limitations

**FIGURE 1:**
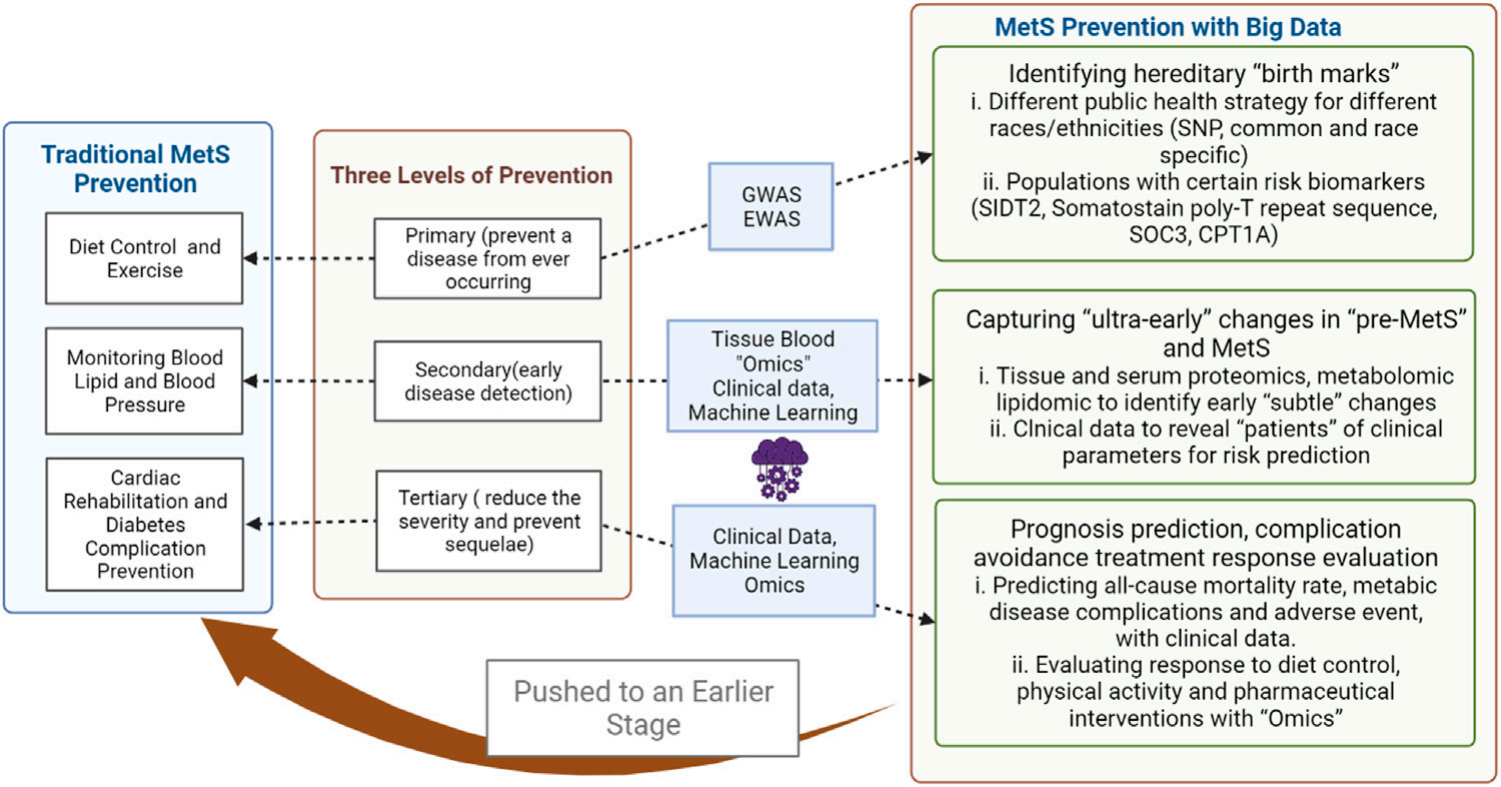
Conceptual framework of prevention comparison on metabolic syndrome (MetS).

“Big data” approaches harness the power from a large amount of data acquired from genetic to clinical levels, with high-throughput technologies and data mining technologies, changing the basic understanding of MetS prevention at every step (Figure 1). Novel single biomarkers for risk evaluation and novel patterns of markers for early detection of disease occurrence and progression could offer new options for health care management at both preventive and clinical medicine levels. However, with the current development, many problems still await solutions.

Information leak is an essential challenge of “big data” research, whether with “omic” studies or data mining of clinical data, causing a significant violation of privacy. Many technologies are currently adopted for the security and privacy of big healthcare data. These technologies would fall into authentication, encryption, data masking, access control, monitoring, and auditing. However, even with these measures, information leak is still not a rarity. Such as for “omics” data, there is a leaking risk even with genomic deletions from signal profiles, but studies have focused on better ways of data sanitization for protection (Harmanci and Gerstein, 2018; Gürsoy et al., 2020). Another challenge is with clinical data mining. For studies using health care databases, information acquired could be flooded with confounding factors without good documentation, and in many situations, missing data could be problematic. For these challenges, approaches to coping with confounder control such as causal graphs and variable selection, and strategies for substituting missing data such as machine learning tools of generative adversarial imputation nets have been studied (Brookhart et al., 2010; Dong et al., 2021).

The most important current problem for bringing “big data” approaches into the practice of MetS prevention is the lack of a unified protocol for the integration of data from different dimensions. Indeed, both multi-omics data from MetS research and automation in data mining for clinical data are just beginning. With limited understanding of the heterogeneity of MetS, many findings and evidence remain in dispute, unable to directly generate prevention recommendations, and this review is narrative in nature, with potential bias in interpretation of the field.

## Conclusion

We have been seeing an acceleration of enrichment in this realm in recent years. Many “omics” and clinical databases still await standardization, which could lower the technical threshold for its integration. Future development in this field could breach the barrier between dimensions of data and newer patterns could be discovered with more versatile analytic tools, enabling more specific preventive strategies for MetS.
